# Preparing facilitators to use and adapt mathematics professional development materials productively

**DOI:** 10.1186/s40594-017-0089-9

**Published:** 2017-11-23

**Authors:** Jennifer Jacobs, Nanette Seago, Karen Koellner

**Affiliations:** 1 Institute of Cognitive Science, University of Colorado Boulder, UCB 594, Boulder, CO 80301 USA; 2 WestEd, 730 Harrison Street, San Francisco, CA 94107 USA; 3 Hunter College, The City University of New York, 695 Park Ave, Ste 913W, New York, NY 10065 USA

**Keywords:** Professional development, Mathematics education, Facilitator training

## Abstract

**Background:**

Determining whether a professional development program can be enacted with integrity in different settings and by different facilitators is critical to understanding efficacy. In this paper, we describe the two-stage preparation process of a facilitator as she prepared to use and adapt the highly specified Learning and Teaching Geometry video-based professional development materials with fidelity. The latter stage of the preparation process involved a rehearsal, during which the research team used two instruments to measure fidelity.

**Methods:**

Two existing instruments were used to explore fidelity through different lenses, including timing and modification of activities and learning goals.

**Results:**

Results from both fidelity instruments indicate that the facilitator used the materials as intended by the developers. However, these instruments did not capture important information regarding modifications the facilitator made, including timing and content-focused adaptations.

**Conclusions:**

Suggestions are made with respect to measuring fidelity, preparing facilitators, and supporting productive adaptations.

## Background

### Facilitating professional development

**Fig. 1:**
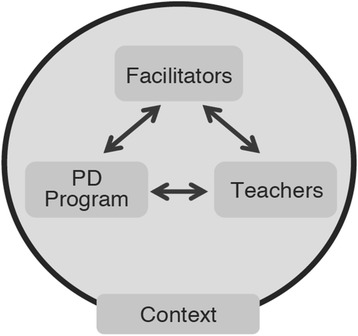
Elements of a professional development system (Borko 2004)

Professional development (PD) for mathematics teachers is central to efforts to improve classroom instruction and student learning, both in the USA and internationally. Building on classroom research that highlights the interaction between the curricular materials, teachers, students, and the local context (Ball and Cohen 1996; Cohen et al. 2003), PD has correspondingly been described as consisting of four key elements: the professional development program, teacher participants, the facilitator, and the local context (Borko 2004; Knapp 2003; see Fig. 1). Unpacking the interactive and reciprocal relationships between these elements allows us to better understand how particular PD programs work and what makes them more or less effective with respect to their learning goals.

Important research has been conducted on what leaders of mathematics PD need to know and do to be effective facilitators (Elliott et al. 2009; Le Fevre 2004). For example, Carroll and Mumme (2007) proposed that facilitators should be equipped with detailed knowledge of the subject matter, information about the teachers they will be working with as well as the students of those teachers, knowledge of teaching both children and adults, and knowledge of how to use the PD materials to create a productive learning environment. Borko et al. (2011b) suggested that leaders of mathematics PD need to be especially well versed in three central facilitation practices: (1) engaging teachers in productive mathematical work, (2) leading discussions about student reasoning and instructional practices, and (3) building a professional learning community.

Other researchers have documented specific facilitation moves that contribute to the effective use of video-based PD programs. In such programs, conversations around the video are considered central to deepening teachers’ noticing and analysis of critical issues related to mathematics teaching and learning, ideally leading to more reflective classroom practice (Sherin and van Es 2005; Star and Strickland 2008; van Es and Sherin 2010). A number of researchers are beginning to offer frameworks to characterize the skillful facilitation of video-based discussions in PD workshops (Zhang et al. 2011; Borko et al. 2014a, b; van Es et al. 2014). For example, van Es et al. (2014) identified facilitation practices that engage mathematics teachers in substantive talk about video. First, effective facilitators orient their participants to the video analysis task at hand, for example by asking questions that enable teachers to readily enter into the conversation. Then, successful facilitators maintain both an inquiry stance and a focus on connection between the video and the mathematics content. Lastly, expert facilitators ensure that the discussion is a collaborative effort in which all participants are engaged and offer a variety of perspectives.

### Facilitating highly specified PD programs

PD programs vary according to their focus, duration, goals, and resources, among other things. Previously, we have argued that PD programs can be understood as falling on a continuum from highly adaptive to highly specified (Borko et al. 2011a; Koellner and Jacobs 2015). The degree to which programs are adaptive or specified offers some general insights regarding their expected facilitation. Highly adaptive programs are designed to be readily responsive or adapted to the local context. Facilitators are likely to have a relatively strong voice in setting the broad components of adaptive PD, including determining the activities that teachers will engage in and defining the structure of their engagement. By contrast, highly specified programs are intended to support a particular learning environment with predetermined goals, activities, and resources. Facilitators of highly specified PD programs are less likely to select activities; rather, they must become familiar with the tasks and structures provided by the PD.

Successful PD programs include enough flexibility so that they are relevant and responsive to the local context, allow key stakeholders to play a role in decision-making, and encourage participants to take ownership of their learning (Coburn 2003; Darling-Hammond and McLaughlin 1995). Facilitators of specified PD programs are further charged with adhering to the specifications and critical features most central to the PD, using the provided resources in the manner intended by the designers, and understanding when and how to make appropriate adaptations.

In research on classroom curriculum there is a distinction between the “formal curriculum,” the “intended curriculum,” and the “enacted curriculum” (Remillard 2005; Tarr et al. 2008). The formal curriculum refers to that written by textbook publishers or outlined in school policies. The intended curriculum refers to teachers’ plans and aims for using the formal curriculum. The enacted curriculum refers to what teachers actually do with the curriculum in the classroom with their students. There is little debate that, as Remillard (2005) put it, teachers are “active users of curriculum materials and shapers of the enacted curriculum” (pg. 215).

Differences between the formal curriculum and the enacted curriculum, in particular, represent the space in which teachers made decisions regarding fidelity and adaptations (Heck et al. 2012). Brown et al. (2009) provided further depth to this line of reasoning about curriculum enactment and fidelity by pointing out that measuring “coverage” of the curriculum is much less relevant to determining fidelity than documenting students’ “opportunities to learn” afforded by the teacher’s enactment relative to the formal or intended curriculum. Extrapolating from curriculum research to the field of PD, opportunities for teachers to learn based on the facilitator’s implementation are at least as important (if not more so) than precise coverage of the given materials.

### Fidelity and adaptation

According to O’Donnell’s (2008) review of the literature, although there are multiple definitions of fidelity of implementation, they are generally quite similar. For our purposes, we define fidelity as the degree to which a facilitator maintains the integrity of the “formal” PD program in their implementation (Borko 2004). When using a highly specified PD program, fidelity entails carrying out the PD in a manner that matches the core activities and learning goals as explicated in the existent PD materials and facilitation resources.

There is some debate regarding the relationship between fidelity and adaptations; however, we side with those who argue that adaptations to any PD program will be necessary to accommodate the unique needs and interests of particular groups of participants (Dane and Scheider 1998). In fact, highly specified PD materials can readily support the coexistence of both implementation fidelity and productive adaptations (Seago 2007). By clearly articulating the recommended workshop activities and facilitation strategies, along with the underlying intentions of the PD, highly specified materials enable informed adaptation decisions that are consonant with fidelity to the designers’ intentions. Seago (2007) proposed that PD facilitators’ adaptations can be categorized using a continuum that includes productive adaptations, no impact adaptations, and fatal adaptations. As long as facilitators make implementation decisions that are productive or have no impact on the participants’ intended experience of the PD, their enactment can be described as maintaining fidelity. Only fatal adaptations adversely impact fidelity by seriously undermining the core principles and goals of the PD.

Mumme et al. (2010) further argue that the need for adaptations to PD programs can arise from external constraints, situational factors, or the facilitators’ knowledge or beliefs. Although this research highlights the range of factors that can influence facilitation choices, preparing PD leaders so that they have a strong knowledge base of the critical features of the program can help to ensure that their adaptations are productive and match the vision of the PD developers. Particularly in the case of highly specified materials, facilitators need to have a thorough understanding of the central learning goals in order to make appropriate “in the moment” adaptations when leading activities and conversations. For example, when leading a discussion, facilitators must have the requisite knowledge to direct teachers’ attention to the critical ideas and topics as intended by the activity (Lesseig et al. 2016).

### Preparing knowledgeable facilitators

Facilitating professional development is ambitious and challenging work. Facilitators enter this work with a variety of professional backgrounds and a range of experiences supporting student and/or adult learning. Even facilitators who have previously led workshops must become familiar with the characteristics, processes, and intentions inherent to any PD program that is new to them. Borko et al. (2014b) proposed that facilitators of mathematics PD draw on their “mathematical knowledge for professional development” as they lead workshops. Building from Ball et al. (2008) construct of mathematical knowledge for teaching, mathematical knowledge for professional development encompasses the specialized content knowledge and pedagogical content knowledge that is required of PD leaders. Specialized content knowledge, in this case, includes a sophisticated understanding of the mathematical concepts and relationships intended to be covered during the PD. Pedagogical content knowledge includes the ability to engage teachers in purposeful activities and conversations about those mathematical concepts and relationships and to help teachers gain a better understanding of how students are likely to approach related tasks. Additionally, mathematical knowledge for professional development includes an understanding of how to establish and maintain a professional learning community in which teachers work together productively and collaboratively. Overall, as supporters of teacher learning, facilitators must hold a deeper and more sophisticated knowledge base than the adults they work with, just as teachers must hold a deeper and more sophisticated knowledge base relative to their students.

Facilitators’ mathematical knowledge for professional development likely has a strong connection to their ability to enact a given PD program with fidelity. If a facilitator deeply understands the mathematical content, is knowledgeable about how to work with teachers around this content, is familiar with the appropriate use of the PD resources, and understands the distinction between productive and fatal adaptations associated with a particular program, they will be better equipped to lead the PD with integrity to its core goals and intentions (Even 1999; Lesseig et al. 2016; Mumme et al. 2010; Remillard and Geist 2002). Gaining the requisite knowledge to become an effective facilitator is likely to be a relatively lengthy, socially constructed process, involving in-depth study and disciplined inquiry (Elliott et al. 2009; Jenlink and Kinnucan-Welsch 2001). As Zaslavsky and Leikin (2004) argue, the construction of mathematics teacher educator’s knowledge is a complex process that typically involves facilitators interacting and learning from both “educators of teacher educators” and teachers.

Just like classroom teachers, facilitators need focused support prior to using a formal PD program to ensure that they can effectively pursue opportunities to unpack and build on teachers’ ideas in line with the program’s goals (Remillard and Geist 2002). Providing sustained and focused preparatory experiences for facilitators—such as cooperative planning meetings and facilitation rehearsals—helps them become confident, proficient, and flexible in their role and promotes their ability to make skillful facilitation moves as they are leading PD workshops (Borko et al. 2014a; Santagata 2009; Zaslavsky and Leikin 2004).

Even (1999) emphasized the importance of holding frequent planning meetings with facilitators who are learning a new mathematics PD program in order to develop their knowledge, leadership skills, and create a professional reference group. Even argued that such meetings are “crucial to the development of a common vision and a feeling of shared ownership” (pg. 20). Especially when the PD is highly specified, careful study of the provided facilitation resources along with guidance from knowledgeable others is likely to help the novice facilitator develop a detailed understanding of the program and acquire a sense of which aspects of the mathematical and pedagogical storyline should be maintained to ensure productive adaptations that maintain fidelity (Heck et al. 2012).

Based on their efforts to prepare 72 facilitators to lead mathematics PD, Lesseig et al. (2016) developed a set of design principles to guide others engaged in this type of preparatory work. Specifically, they recommend focusing on teacher learning goals, providing opportunities for facilitators to expand their specialized content knowledge, and using video or other artifacts of practice to generate in-depth discussion and reflection. Certainly structured planning meetings of this sort are essential to ensure that facilitators are adequately prepared to effectively lead PD. We propose that opportunities to “practice” facilitation techniques, especially when using a new PD program, are a beneficial next step.

Lampert et al. (Lampert 2010; Lampert et al. 2013) have argued that rehearsals are a particularly powerful tool for the professional preparation of beginning teachers. Engaging in thoughtfully constructed rehearsals can support novice teachers to gain practical experience managing a realistic and intellectually ambitious learning environment. Among other things, rehearsals allow teachers to practice eliciting and responding to students’ ideas in ways that meet defined instructional goals. In addition, feedback and reflection from rehearsals can inform planning strategies and generate new ideas (Horn and Little 2010). Benedict-Chambers (2016) proposed that rehearsals can be utilized to build beginning teachers’ understanding of the complexity of classroom interactions and to help them notice and attend to critical features of instruction. We suggest that these same benefits apply to novice PD facilitators or those learning an innovative PD program. For mathematics PD leaders, participating in one or more rehearsals as part of the preparation process provides an opportunity to gain mathematical knowledge for professional development that can directly impact fidelity and promote productive (or no impact) adaptations.

### The Learning and Teaching Geometry PD program

Our paper is based on an efficacy study of the newly developed Learning and Teaching Geometry (LTG) video-based mathematics professional development (Seago et al. 2017), including whether it produces a beneficial impact on teachers’ mathematics knowledge, classroom teaching practices, and their students’ knowledge in the domain of geometry. The LTG PD program supports up to 54 h of guided professional learning for secondary mathematics teachers. The overall goal of the LTG PD is to improve the teaching and learning of mathematical similarity based on geometric transformations, a topic that has taken on increased importance with the U.S. Common Core State Standards for Mathematics (Seago et al. 2013). The materials follow a learning trajectory that is designed to enrich teachers’ mathematical knowledge for teaching as well as their ability to support students’ understanding of congruence and similarity in alignment with the Common Core.

### The role of video in the LTG PD

The LTG project used a design research approach to create the video-based in-service PD program. A central focus of the program is on video clips from a wide variety of classroom lessons, intentionally sequenced to follow a mathematical trajectory. In total, the program includes over 50 video clips, selected from real classroom footage of mathematics lessons across the USA. The clips offer a window into a variety of issues related to content, student thinking, and pedagogical moves. By focusing on classroom video that represents a range of grade levels, geographic locations, and student populations, the program provides insight into what an emerging understanding of similarity looks like as well as specific instructional strategies that can foster this understanding (Seago et al. 2010).

In the LTG materials, video viewing is intentionally sequenced such that it occurs between designated activities. This “video in the middle” design means that video is sandwiched between activities such as mathematical problem solving and pedagogical reflection (Seago 2016). These three elements (pre-video activities, video viewing, and post-video activities) taken together comprise a videocase. Although it is situated “in the middle,” the video clip is in fact the primary ingredient in the design, serving as a focal point of the videocase. Once the video clip has been selected, activities are designed around it to ensure that teachers will engage deeply with the targeted mathematics content, instructional components, and/or student thinking depicted in the clip. The activities surrounding the video also serve as transitions to and from other activities (or video cases) within a given PD session.

Typically, before watching the video, teachers work on and discuss the same problem they will see students working on during the video clip. They might make predictions about how students will solve the problem or what mistakes they might make. After watching the video, the teachers consider the mathematical and pedagogical issues that were brought up by the clip and then reflect on how those issues relate to their own classroom instruction. Specifically, they analyze student’s methods and thinking and the mathematical content that emerges within the teacher and student interactions. In addition, they use evidence from the transcripts to back up claims they make. Taken together, placing video “in the middle” of other PD activities promotes conversations about critical issues related to teaching and learning geometry (Seago 2016). In addition, by situating the PD in actual classroom practice, video helps motivate discussions of how teachers can apply their newly gained insights from the PD to make improvements in their own lessons.

### Facilitation resources

As a highly specified model of PD, the LTG PD program contains stated learning goals^1^, explicit design characteristics, and extensive resources for facilitators. Facilitation resources include a detailed agenda for each workshop session, PowerPoint slides, video clips and transcripts, lesson graphs^2^, mathematical tasks and other handouts, a Field Guide to Geometric Transformations, Congruence, and Similarity, interactive computer applets, embedded assessments, and a comprehensive Facilitator’s Guide. These resources aim to support facilitators in maintaining the intended mathematical and pedagogical storyline of each session while necessarily adapting the materials to unique groups of participants and their learning and working contexts.

Likely the most important LTG PD facilitation resources are the session agendas. These agendas are intended for the facilitators (not the participants) and contain critical information needed to lead a given session. Each agenda lists the main mathematical focuses (e.g., goals) of the session, and for every activity in the session, the agenda includes a detailed description, a suggested time allotment, the necessary materials, guiding questions, and extensive notes (e.g., further description of the purpose, suggestions for carrying out the activity, optional guiding questions, mathematical support, cautionary notes). Having session agendas with predictable structures is intended to support facilitators in using the materials with integrity to the LTG PD goals and principles while also shaping the parameters for both adherence and flexibility.

### Demonstrated effectiveness

Preliminary research on the effectiveness of the LTG PD program, which took place concurrent with its development, offers evidence of the promise of the program to impact teacher and student learning. A portion of the LTG PD materials, the Foundation Module, was piloted in eight sites throughout the USA in order to generate both formative and summative evaluation data. Based on data collected from this pilot, the LTG PD program was shown to lead to significant gains in teachers’ geometry content knowledge, along with the knowledge to effectively convey that information in the classroom. On a content knowledge assessment, the treatment teachers demonstrated an average gain of almost 9 percentage points from pretest to posttest, which was significantly higher than the comparison teachers’ average gain of less than 2 percentage points. Similarly, on assessments embedded within the PD that addressed content and pedagogical content knowledge, the treatment teachers significantly improved on five of the six questions, whereas the comparison teachers did not show significant improvement on any question (Seago et al. 2014).

There is also initial evidence that teachers’ engagement in the Foundation Module can lead to significant increases in their students’ knowledge. Specifically, on an assessment closely tied to the mathematics content in the PD materials, the average pretest–posttest gain for students of treatment group teachers was more than 6 percentage points higher than that for students of comparison group teachers (Seago et al. 2014). The demonstrated gains by both teachers and students suggest that the LTG PD program helps to address the pressing need to provide PD opportunities that improve the learning and teaching of mathematics content explicitly targeted by the Common Core State Standards for mathematics (Marrongelle et al. 2013; Sztajn et al. 2012).

### The Learning and Teaching Geometry Efficacy Study

The LTG Efficacy Study aims to further explore the effectiveness of the LTG PD program using a randomized, experimental design. The sample is comprised of 108 mathematics teachers (serving grades 6–12) and their students. Approximately half of the teachers were randomly assigned to take part in the LTG PD in the first intervention year and half will take part in the second intervention year. The intervention consists of the entire LTG PD program, including a 1-week summer institute and 4 days of academic year follow-up sessions beginning in Summer 2016.

As a phase two research endeavor, a primary goal of the LTG Efficacy Study’s central goal is to determine whether the PD program can be enacted with integrity in various settings by a facilitator who was not a developer of the materials (Borko 2004). Most commonly efficacy studies examine the degree to which an intervention has the desired effect under ideal circumstances (O’Donnell 2008), such as utilizing a facilitator with extensive content knowledge and training. As previously noted, in an earlier pilot study that took place as the LTG materials were being developed and revised, facilitators with varying backgrounds from across the USA led workshops in eight sites. During observations of these workshops, project staff noticed a large degree of variance in implementation and made note of facilitator knowledge and skills that appeared likely to correspond to higher levels of effectiveness and fidelity. Specifically, facilitators with strong content knowledge and experience leading mathematics PD appeared to be the most successful in terms of both supporting learning and using the materials in a manner consistent with the expectations of the development team. Based on these experiences, Hannah was selected as the facilitator for the LTG Efficacy Study. Hannah had an exceptionally strong background in the mathematics content (including authoring textbooks) along with prior experience designing and delivering PD institutes focused on similar content. However, Hannah had never facilitated using the LTG PD program, nor had she viewed the materials prior to the start of the LTG Efficacy Study. The co-PI’s (i.e., the authors of this paper) worked with Hannah for an extended period of time to prepare her to lead the LTG PD, as described below.

In efficacy studies, it is essential to demonstrate that a facilitator can implement the PD program with fidelity, in order to argue that any demonstrated impact (or lack thereof) is a true reflection of the PD program and cannot be attributed to implementation failure (Carroll et al. 2007; Raudenbush 2007). Therefore, we systemically documented Hannah’s fidelity of implementation as part of the preparation period (during a facilitation rehearsal). In this paper, we describe both the preparation period and Hannah’s fidelity ratings using existing measures of PD implementation fidelity. We build on these experiences to offer some general conclusions and suggestions regarding the preparation of PD facilitators and the measurement of fidelity.

## Methods

First, we describe how the facilitator, Hannah, prepared to implement the LTG PD program with fidelity as part of the LTG Efficacy Study. This preparation took part in two stages, over an 8-month time period. In the first stage, Hannah individually studied the PD materials, session by session, and then discussed each session with the co-PIs/PD developers. In the second stage, Hannah conducted a rehearsal facilitation of the materials, which was observed by the co-PIs in order to document fidelity and note adaptations. In the sections below, we describe each of these stages in more detail. We also present information on the measurement of fidelity and classify some of the ways in which Hannah adapted the materials during the rehearsal.

### Stage I: individual study of the materials and advice seeking

The co-PIs worked closely with Hannah to support her understanding of the LTG PD program, address questions and concerns, ensure a common vision, and document the preparation process. Hannah met via video conference with the co-PIs several times a month for approximately 8 months, covering roughly two PD sessions per month. Prior to each meeting, Hannah independently studied a specified portion of the materials (generally one PD session) by going through all of the provided facilitation resources, working out the mathematics problems, watching the associated video clips, and thinking carefully about recommended facilitation strategies. She kept written notes about her ideas and questions and shared those notes with the co-PIs prior to our meetings. The co-PIs audio taped and took notes during each meeting, documenting the manner in which Hannah’s concerns and questions were taken up and addressed.

A central component of Hannah’s individual study was her attention to the specifics in each session agenda. Hannah was highly attentive to the nature of each workshop activity, including the timing and purpose of the activity, the mathematical goals, and the suggested probing questions. Hannah made notes for herself in the margins of the agendas, which she later drew on when facilitating. These notes included topics that she wanted to be sure to give enough attention to, questions or concerns she had about how teachers would respond to an activity or probe, and additional questions to ask or mathematical connections to make if appropriate. Hannah added to or revised her notes based on conversations with the co-PIs. Prior to the facilitation rehearsal, Hannah again independently reviewed the session materials and her notes.

### Stage II: facilitation rehearsal and fidelity documentation

In February 2016, Hannah conducted a 2.5-day rehearsal of the first half of the LTG Foundation Module in order to practice using the materials and to determine whether her facilitation was in line with the developers’ intentions. Hannah drew upon her Stage I work and knowledge of the participants to plan and prepare for the rehearsal. The rehearsal was held at the University of Hawaii in Honolulu with eight participants—including five secondary mathematics teachers from the University Laboratory School in Honolulu, HI, and three mathematics educators from the University of Hawaii at Manoa College of Education’s Curriculum Research and Development Group. As a recently retired faculty member of the University of Hawaii and former secondary math teacher at the University Laboratory School, Hannah knew all of the participants. The three co-PIs attended and videotaped the rehearsal. In addition, the co-PIs documented, individually rated, and came to consensus agreement on Hannah’s fidelity of implementation ratings^3^. At the conclusion of each day, the co-PIs informally provided feedback to Hannah and discussed her adaptations.

Fidelity of the rehearsal workshops was analyzed using modified versions of two existing instruments in order to explore fidelity through different lenses, based on the current literature. Both instruments are session-specific, meaning that they take into account fidelity to the written agenda for each workshop session. As such, both instruments had to be modified to incorporate the specific activities or stated goals for each session of the LTG PD program. Together, the instruments capture adherence to the intended mathematical and pedagogical storylines and the degree to which modifications were made.

## Results and Discussion

The first fidelity instrument was created by Horizon Research, Inc. (HRI) specifically for the LTG PD program and was slightly adapted for this study to account for an updated version of the PD materials. This instrument, the LTG PD Session Logging Tool, measures (1) adherence to suggested time spent on each activity category^4^ within a given session and (2) the extent to which individual activities (within each activity category) were modified. When used to measure fidelity during the LTG pilot study, data from the Session Logging Tool was triangulated with facilitator interviews and direct observations of PD workshops. The quantitative information provided by instrument was deemed to be a valid indicator of activity timing and modification.

As shown in Table 1, using the Session Logging Tool, adherence to allotted activity times is rated on a 4-point scale: no time spent on the activity, less time than allotted, about the amount of time that was allotted, and more time than allotted. In her rehearsal of LTG sessions 1 through 4, Hannah spent about the allotted time on at least half of the activity categories. In session 5, Hannah left out three activity categories and spent less than the allotted time on four activity categories. Considering the rehearsal as a whole, Hannah did not cover 9% of the activity categories, she spent either more or less than the allotted time on 44% of the activity categories, and she spent the allotted time on 48% of the activity categories.

**Table 1:** Hannah’s fidelity ratings using the LTG PD Session Logging Tool

	Time spent on each activity category				Extent individual activities were modified	
	No time spent	Less than allotted	About allotted	More than allotted	Not modified	Modified
Session 1 activities	0/8	1/8	5/8	2/8	29/29	0/29
Session 2 activities	0/8	2/8	5/8	1/8	21/21	0/21
Session 3 activities	0/10	2/10	5/10	3/10	18/18	0/18
Session 4 activities	1/9	2/9	5/9	1/9	15/17	2/17
Session 5 activities	3/11	4/11	2/11	2/11	18/18	0/18
Total^a^	9%	24%	48%	20%	98%	2%

^a^Percentages may not total to 100 due to rounding

The extent to which individual activities were modified during the rehearsal was rated on a 5-point scale using the Session Logging Tool, from not modified at all to substantially modified. However, in Table 1, we have condensed this scale because almost all of the activities in Hannah’s rehearsal were implemented as intended and not modified. If an entire activity category was left out of a session during the rehearsal, the individual activities within that category were not rated for modifications. In four of the five sessions, no activities were modified. In one session, 2 (out of 17) activities were modified. Looking across the sessions, 98% of the individual activities (within activity categories that were carried out) were not modified.

The second instrument used to measure fidelity of implementation (FOI) of Hannah’s rehearsal was a modified version of the Teacher Learning Goals instrument. The Teacher Learning Goals instrument is derived from a fidelity instrument created by the Center for Elementary Mathematics and Science Education at the University of Chicago (Century et al. 2008, 2010) and was previously adapted by WestEd’s Linear Functions for Teaching efficacy study (IES# R305A100047). Century et al. (2010) instrument was developed through an extensive and iterative process that included a written materials review, interactions with PD developers, a review by users, and piloting and field testing all components of their instrument with multiple math and science instructional materials programs, leading them to argue that it is valid and appropriate for wide ranging educational programs.

In contrast to the Session Logging Instrument which focuses on timing and modifications of activities, the Teacher Learning Goals instrument focuses on goals. The instrument comprises the critical components of a given PD program, which “are the operationalizations of developer’s beliefs; they are the measurable elements of the intended program model and thus the primary focus of FOI measurement” (Century et al. 2008, pg. 4). Our modified Teaching Learning Goals instrument examined whether Hannah maintained fidelity to the LTG PD program’s stated mathematics goals and mathematical knowledge for teaching (MKT) goals within each session of her rehearsal.

As shown in Table 2, the Teacher Learning Goals instrument utilizes a 3-point rating system to score the facilitator’s fidelity. The rating system takes into account adherence to learning goals as well as the appropriateness of any adaptations. For example, a rating of high fidelity (3) indicates that the facilitator both addressed the stated goal and made appropriate adaptations as needed. Each of the three co-PIs individually rated sessions 1–5 of the rehearsal and then discussed any discrepancies until they came to agreement.

**Table 2:** Scoring guide for the Teacher Learning Goals instrument

Score	Description	Rating
Low fidelity	Facilitator does not adhere to the goal and/or does not adapt to the participants’ or context in any way.	1
Medium fidelity	Facilitator sometimes adheres to the goal and/or sometimes adapts to the participants’ or context.	2
High fidelity	Facilitator adheres to the goal and adapts to participants’ and/or context as needed, while maintaining the integrity of the PD design.	3

Table 3 shows Hannah’s fidelity ratings using the Teacher Learning Goals instrument for each goal within all five sessions that she facilitated. In all cases, Hannah’s facilitation was judged to be of high fidelity for each goal, with the exception of one goal in session 5 (“planning for classroom use of the Geometric Transformations Workouts”) that was intentionally left out of this rehearsal. In other words, Hannah led each LTG PD session in a manner that was consistent with the developers’ specified goals for that session.

**Table 3:** Hannah’s fidelity ratings using the Teacher Learning Goals instrument

Teacher Learning Goals				
	Focus on Math goals	Rating	Focus on MKT goals	Rating
Session 1	Exploring congruence with and emphasis on geometric transformations	3	Examining students’ arguments, explanations, and understanding of congruence	3
	Examining the mathematical meaning of same	3	Building on students’ mathematical meaning of sameness	3
	Examining the definitions and properties of translation, rotation, and reflection	3	Moving towards more precise language around congruence	3
	Understanding translation	3		
Session 2	Distinguishing and representing static and transformations-based conceptions of similarity	3	Representing student’s methods; classifying students’ definitions of similarity as static or transformations-based	3
	Introducing within and between figure relationships	3	Determining students’ conceptions of similarity, including their attention to relationships within and between figures	3
	Examining why congruent figures are similar	3		
	Understanding rotation	3		
Session 3	Defining the relationship between dilation and similarity	3	Interpreting and representing students’ mathematical ideas around dilation	3
	Examining dilation as a tool for testing whether figures are similar	3	Choosing mathematical representations for conveying content to students	3
	Understanding reflection	3	Examining teacher and student language	3
Session 4	The preservation of angles through dilation	3	Interpreting students’ mathematical arguments about dilation and dilated figures	3
	The effect of moving the center of dilation	3	Teaching dilation: appropriate definitions, language, and visuals	3
	Similarity of circles	3		
	Using technology to explore and conceptualize the dynamic nature of dilations	3		
Session 5	Dilation preserves angles; corresponding angles are congruent in similar figures	3	Exploring the importance of precise language	3
	Measures of corresponding lengths in similar figures are proportional, even in irregular figures	3	Examining students conceptions of the connections between dilation, proportional reasoning and preservation of angles	3
			Planning for classroom use of the Geometric Transformations Workouts	N/A

### Summary of the fidelity findings

Comparing the two fidelity instruments used in this study, the LTG PD Session Logging Tool indicates somewhat lower fidelity of implementation ratings compared to the Teacher Learning Goals instrument. In particular, results from the Session Logging Tool indicate that Hannah made timing adjustments in every session. These adjustments were more or less evenly split between adding more time to an activity category or taking less time to complete an activity category. In a few cases (in sessions 4 and 5), activity categories were left out of the session. The activity categories that were left out of the rehearsal focused on using the PD materials in the teachers’ classrooms (which was not appropriate for this group of teachers, given the specifics of their secondary mathematics curriculum), completing a set of mathematics tasks (which was deemed to be below their mathematical skill level), and reflecting on the last session (instead they reflected on the PD as a whole).

The Session Logging Tool captures timing as a component of fidelity without taking into account the appropriateness of any time adjustments. This type of fidelity measurement does not capture the degree to which modifications were made “with integrity” to the designers’ intentions, only to the degree to which they were made. In all cases, the decisions that Hannah made to spend more or less time on a given activity category, or to leave out an activity category, were informally discussed by the raters and deemed to be consonant with the designers’ intentions. Additionally, the instrument does not account for activities that a facilitator may add to a given session. For example, in session 5, Hannah elected to include an activity from a later session in the LTG PD materials since her group would not be doing that session and she felt the activity would be useful for their learning.

Both the Session Logging Tool and the Teacher Learning Goals instrument examine the facilitator’s modifications within a session. However, the Session Logging Tool focuses on smaller units (activities), whereas the Teacher Learning Goals instrument focuses on more broadly defined goals. Additionally, the Session Logging Tool captures the degree to which there are modifications within an activity, whereas the Teacher Learning Goals instrument simultaneously captures modifications and their appropriateness. Using both of these instruments, Hannah’s rehearsal of the LTG PD program can be characterized as high fidelity, with appropriate adaptations made as needed based on the PD context. However, neither instrument probed deeply into the specific nature of Hannah’s adaptations, prompting us to qualitatively take up such an endeavor as discussed in the next section.

### Hannah’s adaptations: some examples

As part of a thorough examination of fidelity of implementation, it is helpful to consider examples of how and why facilitators modify aspects of the target program to fit the needs of the participants and their unique context. Exploring these adaptations helps to determine whether they should be classified as productive, no impact, or fatal. In the case of Hannah’s rehearsal workshops, we categorized her adaptations as falling into two overarching categories: timing and content-focused. Below, we provide specific examples and a broad classification of each type of adaptation.

### Timing adaptations

Hannah’s rehearsal was scheduled such that she only had time to go through half of the LTG Foundation Module (i.e., to facilitate five out of ten sessions). Hannah had carefully studied all ten sessions in phase 1 of the preparation process, and because the sessions are intentionally sequenced to follow a specific mathematical trajectory, she knew it was important to go through them in order. Therefore, Hannah planned in advance to use only the first five sessions with her participants. However, Hannah was familiar with the other five sessions and towards the end of the rehearsal she elected to bring in an activity from a later session that fit the participants’ mathematical interests and supported elements of their curriculum.

Another timing adaptation Hannah made was to modify the amount of time spent of specific workshop activities. In total, Hannah modified the timing of just under half of the activities in sessions 1 through 5, as shown in Table 1. Her modifications were more or less evenly split between taking less time than allocated by the session agenda (20%) and taking more time (24%). On the whole, these modifications balanced each other out and Hannah completed each session within the given time of 3 h. It is informative to look more closely at the types of activities Hannah spent more or less time on—relative to the allotted time—to determine whether she showed a preference for one activity category over another. As Table 4 indicates, there were some patterns in Hannah’s timing decisions. When she allocated more time to an activity, it was most often to a math task, and when she allocated less time to an activity, it was more often to a video discussion. It is possible that Hannah’s strong facility with the mathematics content led her to focus more on discussions of tasks than on the video footage.

**Table 4:** Time Hannah spent on various activities during the rehearsal

	Less time than allocated	More time than allocated
Math tasks	3	6
Video discussions	6	2
Misc (e.g., introducing the lesson, reflections)	2	1
Total	11	9

Hannah’s timing adaptations were judged by the raters to be “no impact” adaptations, using Seago’s (2007) classification system discussed earlier. Hannah was deemed to make appropriate in-the-moment decisions to determine exact amount of time to spent on a given activity using their impression of the participants’ understanding of the content, engagement in the discussion, and overall sense of the session timing. Although facilitators of the LTG PD program are expected to cover all of the activities in their given order, spending more time on some activities and the less time on others within a session is generally considered reasonable and appropriate.

### Content-focused adaptations

Content-focused adaptations refer to additions or alterations that Hannah made to the PD materials in an effort to meet the perceived mathematical learning needs of her participants. During the rehearsal, the participants voiced an interest in exploring the connection between transformations-based geometry and algebraic concepts, such as slope and graphs of linear equations. Making connections between geometry and algebra is an important feature of the LTG PD program, but it is a feature that does not come into sharp focus until the later sessions of the Foundation Module. After the first day of the rehearsal (i.e., after session 2), Hannah made the decision to leave out a few activity categories in sessions 4 and 5 in order to incorporate some activities from session 8 that she was originally not planning to cover. Specifically, the sections of the materials that Hannah left out were making connections to classroom practice in sessions 4 and 5, and one of the math problems in session 5. All of the activities that Hannah left out were mathematically below the ability level of her participants and pedagogically not particularly relevant to their students. Because the rehearsal included only a few teachers who all taught at the same school, this judgment was clear to Hannah as well as to the fidelity observers, thereby leading the raters to classify it as a “productive adaptation”. The rehearsal teachers were a fairly unique group in the sense that they already had extensive knowledge in the basics of geometric transformations, and several were actively teaching this topic to their students across grade levels. Therefore, moving into more challenging content seemed appropriate given their interest, prior knowledge, and time considerations.

Another content-focused adaptation deemed to be “productive” occurred during the discussion of a mathematics task towards the end of session 1 when Hannah introduced the term “inspection method” to conceptually describe one participant’s method for solving the task. Hannah again used this term in session 3 after a teacher explained that he “saw” the solution to a problem based on his basic understanding of mathematical properties and geometric transformations. Later, the term was taken up by other participants, who used it to describe student methods in some of the video clips they watched and to categorize how many students in their own classes work on geometry problems. In their written reflections, several teachers mentioned that they had more appreciation for their students’ visual approaches to math problems. What is interesting about Hannah’s introduction of the term “inspection method” is that the term is not highlighted in the LTG PD materials, nor was it discussed during phase 1 of the preparation period, but it fits readily into the mathematical and pedagogical storyline. Sometimes referred to as “eyeballing,” visual inspection methods rely on carefully studying and reasoning about the visual makeup of an object and are a precursor to more sophisticated mathematical reasoning. By coining a name for this approach, Hannah helped teachers notice its importance in the intended learning trajectory and built on the participants’ ideas to promote multiple mathematical and MKT learning goals.

Hannah’s adaptations were clearly in line with the intentions and goals of the materials, and none would be considered “fatal.” She did not venture far from the provided resources and remained focused on the goals within each session to support participants’ learning. Her experiences preparing to facilitate, combined with her growing understanding of the participants’ mathematical knowledge and instructional environment, appeared to support her ability to actively listen and react to the participants’ ideas, seizing upon “teachable moments” while ensuring the integrity of the LTG PD program.

## Conclusions

Vital to the exploration of a given PD program’s impact is determining whether the program under study is implemented with fidelity. Loucks-Horsley et al. (2003) noted that part of the designers’ job is to ensure PD leaders have the requisite expertise to facilitate the program as intended and are able to respond appropriately to a given group of teachers. In this study, the co-PIs engaged in a sustained preparation period with the facilitator to help her gain the requisite knowledge and skills to use the materials with fidelity and make productive or no impact adaptations as needed. Although we cannot say with absolute certainty that Hannah will always implement the LTG PD program with fidelity, we can say that she has demonstrated the capacity to do so.

In this final section, we consider several issues related to fidelity. First, we explore the connection between fidelity, adaptations, and the improvisational nature of facilitation. Next we consider how fidelity should be measured, particularly in the context of leading a mathematics PD program. Finally, we consider the process of preparing facilitators to lead PD with fidelity and make some conjectures about scaling. Akin to skillful facilitation, our aim is not necessarily to provide answers but rather to bring challenging issues to light for thoughtful consideration.

### Expecting adaptations and ensuring they are productive

Facilitators, like teachers, must make continual, in-the-moment decisions that take into account the interplay between their PD program, the participants, and the situational context. Whether these decisions impact their fidelity of implementation is a critical issue to consider, for example to understand variation across workshops and to gauge the effectiveness of the PD. Although the LTG PD program is a specified model of PD with a pre-defined set of activities and goals, it rests on the foundation that facilitators will need to make decisions regarding adaptations based on the nature of their participants and the circumstances under which they are working.

A number of researchers have argued that classroom teaching can be understood as improvisational performance (Barker and Borko 2011; Borko and Livingston 1989; King 2001; Remillard 1999; Sawyer 2004). In an analogous manner, PD facilitators can be viewed as engaging in disciplined improvisation, a process in which they lead teachers through the framework of a particular program in a way that demands ongoing decision-making and interpretation. Barker and Borko (2011) posit that “effective instruction (i.e., teaching that results in student learning)… requires a teacher’s deft coordination of the complex interactions among herself, students, the content, and the specific community context” (pg. 280). We argue that this statement rings equally true for PD facilitators and furthermore that the “deft coordination of complex interactions” will often require (no impact or productive) adaptations.

Remillard and Geist (2002) studied specific moments in mathematics PD workshops when facilitators must make decisions about how to react to unanticipated conversation paths. The researchers labeled these moments “openings in the curriculum” and suggested that facilitators’ responses draw on their knowledge, beliefs, and understanding of the PD aims. Teacher statements or questions that are especially challenging necessitate adept choices by the facilitator and demonstrate that appropriate adaptations can mitigate tensions in the facilitator-teacher relationship. As facilitators gain more experience, they should become better able to deliberately utilize “openings” or tensions as opportunities to support teacher learning goals. Remillard and Geist (2002) conjecture, “as they learn to examine various responses [to teachers] in light of possible consequences, facilitators will grow increasingly aware of the range of navigational choices available to them and of the connection between those choices and what participants may be learning over time” (pg. 30). Certainly, an area for further study is the relationship between facilitation expertise and productive adaptations.

### How should fidelity be measured?

As Century et al. (2010) have argued, “Given that adaptation happens, a unidimensional view of fidelity of implementation that results in a single score or rating does not accommodate the dynamism of intervention enactment in the real world” (pg. 214). Education researchers interested in measuring fidelity generally report on two broad dimensions: structural and process (Harn et al. 2013; Mowbra et al. 2003; O’Donnell 2008). Structural dimensions of fidelity look at more objective components of the intervention, such as timing and coverage of material. Process dimensions examine more subjective components, such as quality of modifications.

In this study, we measured fidelity using two instruments that, together, incorporate structural and process dimensions. However, the Session Logging Tool placed a larger emphasis on structure, whereas the Teaching Learning Goals instrument placed a larger emphasis on process. Although both of these dimensions yield important insights, we argue that it is especially critical to look at the nature of the facilitator’s adaptations to the PD program and how they relate to the core principles and critical features of the PD (Penuel and Means 2004). By ensuring that the measurement of fidelity includes context-based decisions and adaptations, it is possible to make more meaningful claims about the degree to which facilitators remain true to the goals and intentions of the program, as they navigate the contextual challenges and opportunities inherent in leading PD. We found the Teacher Learning Goals instrument to be especially useful in this regard, but we argue that it did not go far enough supporting the classification of adaptations as productive, no impact, or fatal.

### How can we scale the facilitator preparation process?

We follow the reasoning that, like teachers, even experienced facilitators can benefit from sustained and focused professional learning opportunities, especially when faced with a new PD program that they are charged with implementing with fidelity. Like teachers, facilitators need time to learn and process the new program, and they need time to rehearse and refine their facilitation skills. The two-stage preparation process that we utilized in this study—incorporating first an in-depth study of the PD materials and then a facilitation rehearsal—represents both a systematic and idealized method of working with a single facilitator. We recognize that the conditions of this study are unique and not easily replicable in most cases. But our experiences and observations from engaging in this extended, two-part preparation process leads us to conjecture that a similar process is likely possible, with some qualifications, on a larger scale.

In studies with larger groups of mathematics PD facilitators, researchers have documented the importance of setting aside an extended period of time for planning preparation work (e.g., Borko et al. 2015; Lesseig et al. 2016; Elliott et al. 2009). Across PD programs, there is a need for facilitators to deeply explore the goals, activities, and recommended facilitation strategies and plan for their implementation in an organized manner. We consider this type of planning “stage 1” of the preparation process. Because our Efficacy Study involved only one facilitator, stage 1 took the form of individual study of the PD materials with frequent support from the PD developers. With multiple facilitators, stage 1 could be modified to accommodate collaborative study within a community of facilitators, led by the PD developer or an experienced facilitator.

We further propose that allotting time for facilitation rehearsals as a second component of the preparation process should be seriously considered in future, larger-scale studies of effective PD. Rehearsals ensure that novice facilitators are able to practice using the PD materials in a structured environment, experiment with facilitation moves, and gain confidence in their role as a leader of a given PD program. In addition, rehearsals enable the measurement of fidelity and provide information about whether a facilitator is ready to implement the PD or whether additional training and support is needed. Finally, rehearsals provide an opportunity for facilitators to receive valuable feedback, in particular on the nature and impact of their adaptations, which can lead to more reflective and skillful practice.

Although Hannah’s rehearsal as part of the LTG Efficacy Study took the form of implementing multi-day PD workshops, rehearsals can be carried out in other ways, especially with larger groups of less experienced facilitators. For example, facilitators might break into small groups and rehearse leading a portion of the PD with their peers. Multiple, brief rehearsals followed by periods of feedback, discussion, and structured reflection might prove an effective strategy for achieving fidelity of implementation, particularly when there is less time for the stage 1.

A critical element in organizing the preparation period (both stage 1 and stage 2) is determining which facilitation strategies underlie effective PD workshops and are thus most important for novice facilitators to learn and practice. Facilitation frameworks (such as those provided by Borko et al. 2014a; van Es et al. 2014; Zhang et al. 2011) might prove extremely helpful in this endeavor. These frameworks draw attention to facilitation strategies akin to what has been labeled “high leverage practices” for beginning teachers and provide guidance as to what facilitators should study and rehearse relative to the PD program they are preparing to implement (Ball and Forzani 2011; van Es et al. 2014).

### Final thoughts

Research on preparing and supporting facilitators of mathematics PD is still at a very early stage, but there are meaningful insights to be gained from the more extensive literature on classroom teaching. At the same time, it is important to acknowledge that facilitating a PD program is, in many respects, different from using curricular resources in the classroom. Even PD programs that are highly specified are unlikely to contain as many tools, artifacts, and resources compared to curricula intended for school-aged students. Also, PD programs generally place fewer constraints on facilitators, for example by assuming that adults will play a more central role in shaping their own learning. As such, facilitators are afforded the space to make interpretive moves, which may or may not affect their fidelity of implementation. It is likely that issues involving identity, knowledge, and beliefs will emerge as critical influences on PD facilitators (Gresalfi and Cobb 2011; Le Fevre and Richardson 2002; Stein et al. 1999) and thus should be incorporated in future studies of facilitation.

Another interesting question to consider is whether and how facilitator preparation and fidelity differs depending on the nature of the PD program. For example, do facilitators of specified PD programs require more training than facilitators of adaptive PD programs? We conjecture that the facilitation of both types of programs is equally demanding in preparation and practice, but the nature of the demand differs. In adaptive PD programs, facilitators must select activities that support teacher learning opportunities, which is not a demand faced by facilitators of specified PD programs. However, in specified PD programs, facilitators must grapple with understanding someone else’s workshop design and learning goals. With respect to fidelity, documenting adherence to the designers’ intentions and the nature of adaptations is a more straightforward endeavor for specified programs with explicit goals. At the same time, there is certainly a strong need for facilitators of both types of programs to be well versed in the content and pedagogy associated with effective facilitation. The field would benefit from future research studies that examine the similarities and differences between the demands placed on facilitators of adaptive and specified PD programs and ensuring they are prepared to meet those demands.

## Footnotes

1 The learning goals for the first five sessions of the Foundation Module are shown in Table 3, which is discussed in relation to fidelity ratings later in the paper.

2 A lesson graph is a document that situates a given video clip within the lesson from which it is drawn. A lesson graph concisely represents the full lesson divided into descriptive segments that show the mathematics unfolding as well as the teacher and student interactions.

3 All three authors of this paper rated the fidelity of Hannah’s rehearsal. Two of the authors are also developers of the LTG PD materials.

4 In each session there are activity categories, such as analyzing a set of video clips. Within each activity category there are usually multiple activities, such as watching each video clip, discussing each clip, and comparing the clips.
